# Anti-Obesity and Diuretic Effects of Immature Watermelon Rind Extract in HFD-Induced Obese Mice

**DOI:** 10.3390/nu18010128

**Published:** 2025-12-31

**Authors:** Yun-seong Lee, Ji yong Kim, Sunju So, Bo-Young Lee

**Affiliations:** 1 Nain Healthcare Co., Ltd., 3, Seodong-ro 20-gil, Iksan 54613, Republic of Korea; iyoonseong@daum.net; 2 Department of Food and Biotechnology, Korea University, 2511 Sejong-ro, Sejong 30019, Republic of Korea; greenroad6943@daum.net; 3 Green Road Co., Ltd., 100, Gukgasikpum-ro, Wanggung-myeon, Iksan 54576, Republic of Korea; 4 College of Korean Medicine, Woosuk University, Wanju 55338, Republic of Korea; thtnswn1016@naver.com; 5 Department of Food and Nutrition, College of Agriculture and Food Sciences, Wonkwang University, Iksan 54538, Republic of Korea

**Keywords:** watermelon rind, immature watermelon, anti-obesity, diuretic effect, high-fat diet

## Abstract

**Background/Objectives:** Immature watermelon (WM) rind contains higher levels of citrulline and potassium than mature fruit and may exert diuretic and metabolic benefits. This study aimed to evaluate the anti-obesity and diuretic effects of WM and salt-treated watermelon rind extract (WMS) in high-fat diet (HFD)-induced obese mice, focusing on changes in lipid metabolism, sodium handling, and tissue-level alterations. **Methods:** Citrulline concentrations in WM and WMS were quantified using high-performance liquid chromatography with ultraviolet detection (HPLC-UV). Four-week-old male C57BL/6 mice were fed an HFD for 6 weeks and subsequently administered WM (380 mg/kg) or WMS (380 mg/kg) orally for an additional 6 weeks. Body weight, food intake, organ and fat-pad weights, serum biochemical markers, and sodium (Na^+^) levels were measured. Histopathological analyses of liver and epididymal adipose tissue were performed to assess non-alcoholic steatohepatitis (NASH) scores and adipocyte morphology. **Results:** WM and WMS contained citrulline at levels substantially higher than those reported for mature watermelons. Both treatments significantly reduced body weight, liver weight, and epididymal fat mass compared with the HFD control. Serum total cholesterol and triglyceride levels were lowered in the WM- and WMS-treated groups. Serum Na^+^ concentrations increased by 43.2 ± 7.6% in WM-treated mice and 21.5 ± 6.6% in WMS-treated mice, suggesting enhanced sodium handling. Histological assessment revealed reduced NASH scores and smaller adipocyte sizes in both groups. These improvements are consistent with the known diuretic and metabolic actions of citrulline and potassium. **Conclusions:** WM and WMS exhibit significant anti-obesity and diuretic effects in HFD-induced obese mice. Their combined actions on sodium excretion, lipid metabolism, and adipose tissue remodeling suggest that immature watermelon rind extracts may serve as promising natural agents for preventing obesity and related metabolic dysfunction.

## 1. Introduction

Obesity is classified as a serious medical condition, as it induces various complications such as diabetes, hypertension, cardiovascular diseases, cerebrovascular diseases, arthritis, breast cancer, and colorectal cancer [1]. According to the World Health Organization (WHO), approximately 4 million deaths annually are attributed to complications caused by overweight and obesity. The prevalence of overweight and obesity quadrupled between 1975 and 2016, and it is projected that by 2030, 42% of U.S. adults will be obese. Annual healthcare expenditure for obese individuals has also been steadily increasing [2,3]. Obesity results from an imbalance between energy intake and expenditure, with excess energy stored as fat in adipocytes [4]. Visceral fat accumulation is considered a major contributor to inflammatory diseases and is closely associated with increased blood LDL cholesterol, triglycerides, and reduced HDL cholesterol, thereby heightening insulin resistance and the risk of cardiovascular diseases [5]. Abdominal obesity, particularly subcutaneous fat accumulation, is reported to lead to metabolic disorders, such as non-insulin-dependent diabetes mellitus, hypertension, and dyslipidemia, increasing mortality rates [6]. Obesity can also result from genetic predisposition, endocrine disorders, medication use, lack of exercise, and eating disorders triggered by psychological stress, all of which pose significant social issues [7]. Excessive sodium intake is strongly linked to hypertension, stroke, cardiovascular diseases, and kidney disorders, as supported by numerous studies [8]. Increasing evidence demonstrates that prolonged high sodium intake is associated with adipocyte hypertrophy and increased white adipose tissue mass, even independent of greater caloric intake [9,10]. The 2010 Korea National Health and Nutrition Examination Survey reported that high sodium intake is a characteristic dietary issue among diabetes patients. The average daily sodium intake for Korean adults was 4878 mg, while that for diabetic individuals was 5939 mg [11,12]. Koreans consume excessive sodium compared to other countries, mainly due to dietary habits involving pickled foods like kimchi, soups, stews, and processed foods such as instant noodles [13]. While exercise and dietary modification are fundamental to preventing and treating obesity, lifestyle changes are challenging. Although pharmacological treatments have been proposed as alternatives, they often yield limited efficacy or cause adverse effects [14,15]. Consequently, research on bioactive compounds derived from natural materials with minimal side effects is gaining traction. Watermelon (*Citrullus lanatus*), a member of the Cucurbitaceae family, is a popular summer fruit with high water content. Beyond its consumption as a fresh fruit, watermelon has also been utilized in medicinal applications [16]. It is rich in minerals such as potassium, phosphorus, magnesium, calcium, sodium, and iron, as well as free sugars (fructose, glucose, sucrose) and organic acids (citric, malic, succinic, and fumaric acids) [17]. Watermelon contains citrulline, an amino acid that aids urea synthesis, promoting diuresis and exhibiting potential effects against edema, nephritis, cystitis, urethritis, hypertension, inflammation, and fever [17]. Previous domestic research on Citrullus lanatus (watermelon) has investigated a diverse range of applications and bioactivities. Studies have characterized its volatile aromatic compounds [18], developed functional beverages utilizing natural pigment extracts derived from watermelon flesh [19], and explored its potential as a raw material in the production of fermented alcoholic beverages [20]. Additional investigations have examined juice concentration techniques employing reverse osmosis [21] and analyzed the spatial distribution of sugar content across different anatomical sections of watermelon and melon fruits [22]. The edible flesh of watermelon has been reported to exhibit antibacterial, depigmenting (whitening), and anti-inflammatory activities [23], whereas the rind demonstrates notable antioxidant and depigmenting properties. Moreover, watermelon seeds have been shown to possess antibacterial, depigmenting, and anti-inflammatory effects [24,25]. Recent murine and human studies have advanced the understanding of watermelon species’ anti-obesity potential. For instance, Kang et al. (2024) demonstrated that *Citrullus mucosospermus* extract attenuated weight gain in high-fat diet (HFD)-induced mice [26]. Furthermore, Daughtry et al. (2023) reported that the consumption of blended watermelon led to reductions in BMI and body fat among children with overweight/obesity [27]. Meanwhile, watermelon rind has been shown to contain high levels of L-citrulline and potassium, which may enhance sodium excretion and modulate lipid metabolism [28,29]. Recent studies have highlighted the growing importance of valorizing agricultural by-products by extracting functional compounds from plant materials traditionally discarded as waste [30,31]. Immature watermelons removed during pre-harvest pruning represent a largely unexplored biomass resource, consisting of a pale, undifferentiated mesocarp without red edible flesh.

In this context, the present study investigates the biological activity of extracts derived from whole immature watermelons to enhance resource utilization and reduce agricultural waste. Specifically, we examined immature watermelons culled during production and analyzed the sodium content of their extracts. The purpose of this study was to evaluate whether a mildly salt-treated immature watermelon rind preparation naturally rich in potassium and citrulline could serve as a potassium- and citrulline-enhanced salty food model designed as a partial alternative to conventional salt. Potassium promotes natriuresis by stimulating Na^+^/K^+^-ATPase-dependent renal sodium transport [32], whereas citrulline improves renal microcirculation and enhances nitric oxide-mediated natriuretic responses [33]. Accordingly, sodium ingested within this matrix is expected to be more readily excreted rather than retained, thereby reducing the metabolic burden typically associated with salt intake [34]. In addition, citrulline has been reported to improve mitochondrial function, promote fatty-acid oxidation, and support thermogenic activity [35,36], suggesting potential anti-obesity effects beyond sodium handling. Ultimately, immature watermelon rind extract and its salt-treated may alleviate obesity and metabolic dysfunction induced by a high-fat diet. This research therefore aims to establish immature watermelon rind as a value-added functional material.

## 2. Materials and Methods

### 2.1. Experimental Materials

**Figure 1:**
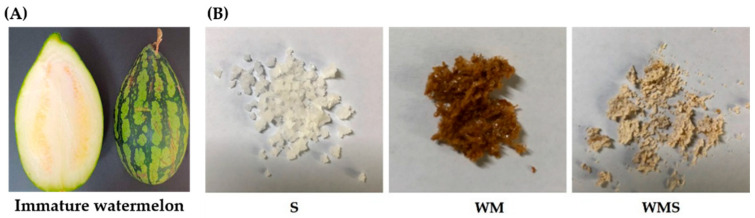
(**A**) Immature watermelon and (**B**) S, salt; WM, immature watermelon extract; WMS, immature watermelon extract treated with salt.

Immature watermelons used in this study were purchased from a local farm in Jeollabuk-do, Korea. The watermelons were sourced from the ‘Urikkul’ cultivar grown in Jeongeup, Iksan, Gimje, and Gochang within Jeonbuk State, South Korea. To ensure consistency in sugar content and moisture levels during processing, we standardized the immature watermelons to a horizontal diameter of 10 ± 1 cm and a vertical diameter of 11 ± 1 cm. Immature watermelons were used at a developmental stage prior to red flesh formation (Figure 1A). At this stage, the mesocarp and epicarp are not yet differentiated; therefore, the whole fruit was sliced and dried as a single integrated material. A total of 30 kg of immature watermelon was washed, thinly sliced, and dried to obtain 2.4 kg of dried material. The dried slices were placed in an extraction vessel, and distilled water five times the weight of the material was added. Hot water extraction was conducted at 100 °C for 3 h and repeated twice. The combined extract was filtered and adjusted to a final concentration of 2–5 Brix. For the preparation of the immature watermelon extract salt (WMS), 1 kg of Korean solar salt (produced in Shinan, Jeollanam-do, Republic of Korea) was gradually added to the immature watermelon extract (WM) while boiling at 100 °C, stirring continuously until fully dissolved. The resulting brine was filtered through a fine mesh (2 mm × 2 mm) to remove impurities and then heated at 200 °C until the salt crystallized. The salt crystals were naturally dried in the shade for over three days and then dried at 60 °C for 4 h using a dryer. After grinding the dried crystals, they were filtered through a fine mesh (2 mm × 2 mm) and further dried at 80 °C for approximately 4 h. The final product, immature watermelon extract salt (WMS), was obtained by re-filtering through the same mesh (Figure 1B).

### 2.2. Citrulline Analysis

Comparing the citrulline content of WM and WMS, component analysis was conducted using high-performance liquid chromatography (HPLC). A standard citrulline solution was prepared at 1000 ppm and serially diluted to concentrations of 0, 20, 40, 60, and 80 µg/mL. Each concentration (10 µL) was injected into the HPLC, and a calibration curve was generated at 207 nm. The chromatographic separation was performed using a YMC-Triart C18 column (250 × 4.6 mm I.D., S-5 μm, 12 nm). The mobile phase consisted of 3 mM phosphoric acid and acetonitrile in a 70:30 ratio, with a flow rate of 0.7 mL/min at room temperature. The calibration curve is represented by the equation

y = 17.22x + 23.72, R^2^ = 0.9986

### 2.3. Experimental Animals

A total of 25 male C57BL/6 mice (4 weeks old) were purchased from Samtako (Osan, Republic of Korea) and used in this study. The animals were randomly allocated into five experimental groups (*n* = 5 per group). The average body weight of the mice was 19.29 ± 0.84 g. Obesity was induced by feeding the mice a high-fat diet (HFD, 60% kcal fat; Samtako, Osan, Republic of Korea), and each extract was suspended in distilled water and administered once daily by oral gavage according to individual body weight to ensure complete dose delivery. The high-fat diet feeding period lasted six weeks to induce obesity, after which all treatments (WM, WMS, refined salt, or water) were administered daily for another six weeks concurrently with HFD feeding. Each group consisted of five mice, which represents a minimal sample size commonly used in rodent obesity studies under the 3Rs principle. The animals were housed under controlled conditions (12 h light/dark cycle, 23 ± 2 °C, 50 ± 10% humidity). The high-fat diet was purchased from Samtako (Osan, Republic of Korea) and its composition is shown in Table 1. All animal procedures were conducted in accordance with the Guide for Animal Experimentation of Wonkwang University and approved by the Institutional Animal Care and Use Committee (IACUC) of Wonkwang University (Approval No. WKU24-44). No predefined inclusion or exclusion criteria were applied during the experiment. No animals or data points were excluded from the analysis, and no attrition occurred during the study. The investigators were not blinded to group allocation during animal allocation, conduct of the experiment, outcome assessment, or data analysis.

### 2.4. Experimental Groups

The mice were randomly divided into five groups as follows: normal control group (CON, fed standard diet and distilled water; DW), high-fat diet control group (HFD-C, fed 60% HFD and DW), HFD with immature watermelon extract 380 mg/kg group (HFD-WM), HFD with salt-treated immature watermelon extract 380 mg/kg group (HFD-WMS), and HFD with refined salt 380 mg/kg group (HFD-S). Each experimental group consisted of five mice, which represents the minimal sample size required to achieve statistical reliability while adhering to the 3Rs principle, as supported by previous rodent studies (Table 2). The oral dose of 380 mg/kg sodium was selected based on human high-salt intake after allometric scaling. Although recommended sodium intake is <2 g/day, actual consumption often reaches 3.0–4.3 g/day (≈50–70 mg/kg in a 60-kg adult) [37]. Using the standard body-surface-area Km conversion factor for rats (≈6.2), this corresponds to ~310–360 mg/kg in rats [38]. Accordingly, 380 mg/kg was chosen as a physiologically relevant high-sodium load that challenges Na^+^/K^+^ homeostasis while remaining within the safe and commonly used dose range for nutritional and toxicological studies. WM and WMS extracts were prepared as freeze-dried powders and reconstituted in distilled water according to body weight extrapolation. The preparations were administered once daily by oral gavage at a fixed volume of 300 µL per mouse (approximately 10 mL/kg), corresponding to a final dose of 380 mg/kg/day, which is consistent with established dosing ranges for phytochemical preparations. All treatments were delivered as aqueous suspensions to ensure accurate and consistent dosing, independent of drinking-water variability. Water intake was recorded separately and expressed as mL/mouse/day.

### 2.5. Measurement of Body Weight and Food Intake

Body weight was measured weekly at a fixed time (10:00 AM) using a digital balance (IB-3100, Innotem, Yangju, Republic of Korea). Food and water intake was recorded twice weekly at consistent times. The food efficiency ratio (FER) was calculated as the weight gain divided by total food intake during the experimental period.

### 2.6. Organ Weight Measurement

Following blood collection, the liver, epididymal fat, and retroperitoneal fat were excised and weighed. The absolute and relative organ weights were calculated. Relative organ weight (%) was calculated using the formula (organ weight/body weight) × 100.

### 2.7. Serum Lipid Profile and Sodium Ion Concentration

After 6 weeks, blood was collected from the retro-orbital vein under isoflurane anesthesia following a 12 h fast and centrifuged at 3000 rpm for 20 min. Serum levels of total cholesterol (TC), triglycerides (TG), high-density lipoprotein cholesterol (HDL-C), and low-density lipoprotein cholesterol (LDL-C) were measured using an enzymatic colorimetric assay (Get Kit, Roche, Mannheim, Germany) on a Modular Analytics device (PE, Roche, Germany). Additional parameters were analyzed using an ion-sensitive electrode analyzer.

### 2.8. Histopathological Analysis

At the end of the experiment, the animals were euthanized using an approved method in accordance with institutional guidelines to ensure death and minimize pain and distress. The liver and adipose tissues were excised, rinsed with saline, and blotted dry. Liver tissues were fixed in 10% neutral buffered formalin, embedded in paraffin using standard procedures, sectioned at 4 μm thickness, and stained with hematoxylin and eosin (H&E). Histopathological observations were conducted at 200× magnification using an Olympus BX50 optical microscope (Olympus, Tokyo, Japan). Nonalcoholic steatohepatitis (NASH) scoring was based on the presence of macrovesicular steatosis, hepatocellular ballooning, and lobular inflammation, mainly observed in zone 3 of the liver. A NASH score ≥ 5 was considered diagnostic of NASH, whereas a score ≤ 2 was classified as non-NASH [39].

### 2.9. Statistical Analysis

All experimental data are presented as mean ± standard deviation (SD). Statistical significance between the groups was assessed using one-way ANOVA, followed by Duncan’s post hoc test using SPSS v.12. A *p*-value < 0.05 was considered statistically significant. Prior to statistical analysis, the data were assessed for normality and homogeneity of variance. All data met the assumptions required for parametric statistical analysis.

## 3. Results

### 3.1. Citrulline Analysis in WM and WMS

**Figure 2:**
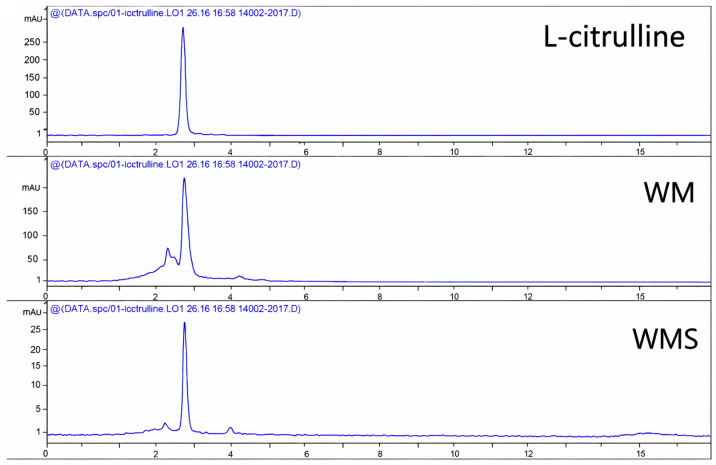
HPLC chromatogram of citrulline in WM and WMS. WM, immature watermelon extract; WMS: salt-treated immature watermelon extract.

Citrulline content in WM and WMS samples was analyzed, with detection occurring at a retention time of 3.029 min. The citrulline concentrations were found to be 7.7 mg/g in WM and 3.1 mg/g in WMS. The results of the analysis of WM and WMS, with a visual representation, are provided in Figure 2.

### 3.2. Change in Body Weight

**Figure 3:**
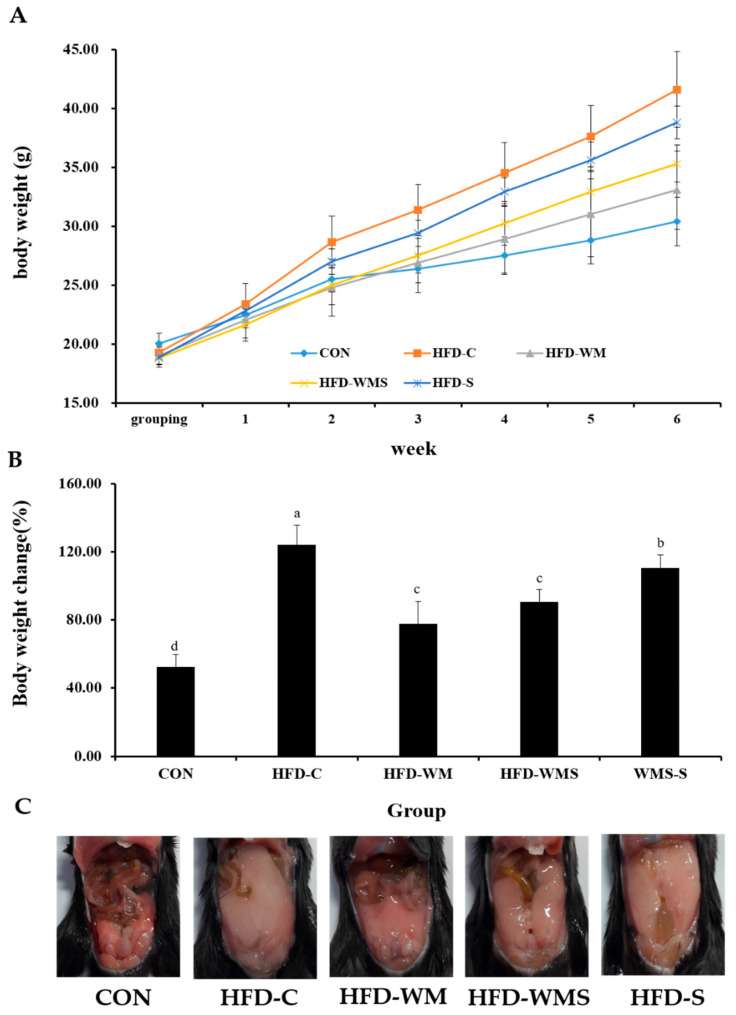
Effects of WM and WMS on (**A**) body weight, (**B**) body weight change, and (**C**) gross appearance of the whole body in C57BL/6 mice obesity model induced by a high-fat diet. CON: Normal Diet + DW; HFD-C: High-Fat Diet + DW; HFD-WM: High-Fat Diet + WM 380 mg/kg; HFD-WMS: High-Fat Diet + WMS 380 mg/kg; HFD-S: High-Fat Diet + Salt. ^a–d^ Means with different superscripts are significantly different (*p* < 0.05) as analyzed using Duncan’s multiple range test.

In a 6-week high-fat diet (HFD) to induce obesity, the administration of the respective test substances led to notable changes in body weight among the experimental groups. No expected or unexpected adverse events were observed during the experimental period. As shown in Figure 3A, both the HFD-WM and HFD-WMS groups exhibited a significant reduction in body weight compared to the HFD control group (HFD-C). By the end of the experiment, the weight gain percentage was 52.4% in the CON group and 124.2% in the HFD-C group, whereas the HFD-WM and HFD-WMS groups showed significantly attenuated weight gains of 79.5% and 88.8%, respectively (Figure 3B). Post-mortem gross morphological observations are shown in Figure 3C. Notable abdominal fat accumulation was observed in all groups except the CON group. However, the extent of fat deposition was visibly reduced in both the HFD-WM and HFD-WMS groups relative to the HFD-C group.

### 3.3. Body Weight Gain, Food Intake, and Food Efficiency Ratio

During the 6-week dietary intervention, data on body weight progression, weight gain, food consumption, and food efficiency ratio were collected (Table 3). Obesity was operationally defined as an increase of ≥20% body weight relative to the standard diet control and a significant increase in epididymal white adipose tissue weight at 6 weeks. In our study, body weight gain was markedly increased in the HFD-C group, with a total 6-week weight gain of 22.35 g, corresponding to a 115.7% increase compared with CON (10.36 g). WM supplementation reduced weight gain to 14.09 g, representing a 37.0% attenuation relative to HFD-C, while WMS reduced gain to 16.51 g (−26.1%). The positive-control group (HFD-S) gained 19.94 g, showing a 10.8% reduction compared with HFD-C. Despite these findings, we recognize that 6 weeks of HFD may produce an early obesogenic phenotype rather than full established obesity. The food efficiency ratio remained consistent across all groups except for the normal control group, with a value of 0.02. The slight, non-significant daily reduction in feed intake observed in the WM and WMS groups may accumulate over time, leading to mild calorie restriction or altered palatability, which could partially explain the observed anti-obesity effect.

### 3.4. Liver Weight and Adipose Tissue Weight

**Figure 4:**
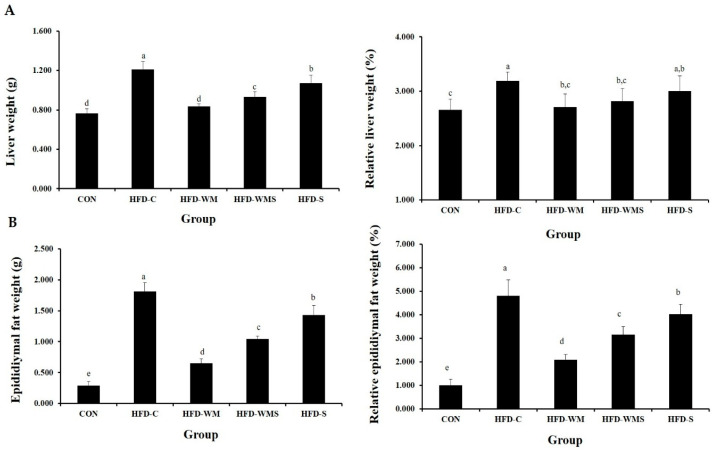
Effects of WM and WMS on (**A**) liver fat weight and relative liver weight, (**B**) epididymal fat weight and relative epididymal fat weight in C57BL/6 mice obesity model induced by a high-fat diet. CON: Normal Diet + DW; HFD-C: High-Fat Diet (60%) + DW; HFD-WM: High-Fat Diet (60%) + WM 380 mg/kg; HFD-WMS: High-Fat Diet (60%) + WMS 380 mg/kg; HFD-S: High-Fat Diet (60%) + Salt. ^a–e^ Means with different superscripts are significantly different (*p* < 0.05) as analyzed using Duncan’s multiple range test.

Liver weights, both absolute and relative (per 100 g body weight), are presented in Figure 4A. HFD feeding markedly increased liver and visceral fat weights compared with the CON group. WM supplementation significantly attenuated these effects. Liver weight was reduced by 31.1% in the HFD-WM group and 23.0% in the HFD-WMS group relative to HFD-C. Epididymal fat mass, which increased more than six-fold in HFD-C compared with CON, was reduced by 64.2% and 42.5% in the HFD-WM and HFD-WMS groups, respectively (Figure 4B). Relative fat mass normalized to body weight also showed consistent reductions, indicating that WM effectively suppresses high-fat diet-induced visceral adiposity.

### 3.5. Triglycerides and Total Cholesterol

**Figure 5:**
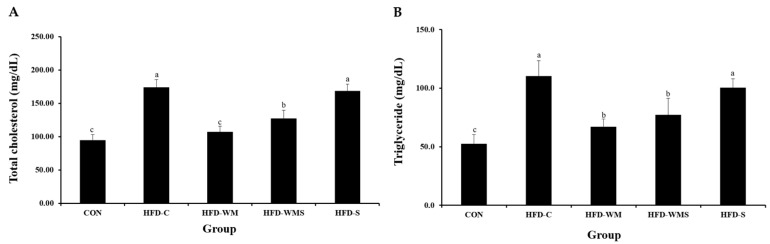
Effect of WM and WMS on (**A**) total cholesterol and (**B**) triglyceride in C57BL/6 mice obesity model induced by a high-fat diet. CON: Normal Diet + DW; HFD-C: High-Fat Diet (60%) + DW; HFD-WM: High-Fat Diet (60%) + WM 380 mg/kg; HFD-WMS: High-Fat Diet (60%) + WMS 380 mg/kg; HFD-S: High-Fat Diet (60%) + Salt. ^a–c^ Means with different superscripts are significantly different (*p* < 0.05) as analyzed using Duncan’s multiple range test.

Serum levels of triglycerides and total cholesterol, the key biomarkers of dyslipidemia and cardiovascular risk, were evaluated (Figure 5A,B). Both parameters were significantly decreased in the HFD-WM and HFD-WMS groups compared to the HFD-C group, implying improved lipid metabolism. In addition to total cholesterol (TC) and triglycerides (TG), detailed values of HDL-C and LDL-C were analyzed. HDL-C levels were significantly elevated in the HFD-C group (128.72 mg/dL) compared with the CON group (75.88 mg/dL, +69.6%). Treatment with WM and WMS markedly reduced HDL-C to 79.76 mg/dL (−38.1%) and 92.76 mg/dL (−27.9%), respectively. LDL-C was calculated using the Friedewald formula. The HFD-C group exhibited a sharp increase (23.20 mg/dL) compared with the CON group (8.40 mg/dL, +176%). WM supplementation lowered LDL-C to 14.00 mg/dL (−39.7%), while WMS reduced it to 19.20 mg/dL (−17.2%) relative to HFD-C. These results confirm that WM supplementation effectively improves HFD-induced dyslipidemia.

### 3.6. Serum Sodium Ion Concentration

**Figure 6:**
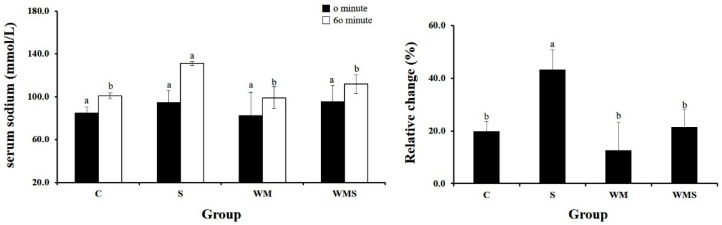
Effect of WM and WMS on the serum concentration of sodium. C, DW; S, Salt 380 mg/kg; WM, WM 380 mg/kg; WMS, WMS 380 mg/kg. ^a,b^ Means with different superscripts are significantly different (*p* < 0.05) as analyzed using Duncan’s multiple range test.

Serum electrolyte concentrations were measured before and 60 min after the administration of the respective samples. As shown in Table 4, the WM group exhibited a 43.2 ± 7.6% increase in serum sodium (Na^+^) levels compared to the S group. In contrast, the WMS group showed a significantly lower increase of 21.5 ± 6.6% (Figure 6). The normal-diet control (CON) group was not included in this subsection because the purpose of Section 3.6 was to evaluate treatment-dependent differences within the HFD-derived experimental groups (HFD-C, HFD-WM, HFD-WMS, and HFD-S), rather than to compare these values with baseline physiological conditions. Baseline comparisons between CON and HFD animals—including body weight, hepatic morphology, adipose histology, and lipid profiles—are already presented in Section 3.1, Section 3.2, Section 3.3, Section 3.4 and Section 3.5. Therefore, the analytical framework in Section 3.6 intentionally used the HFD-control (HFD-C) group as the reference to determine the extent to which each intervention modulated HFD-induced metabolic alteration.

### 3.7. Histological Effects of WM on Liver and Epididymal Adipose Tissue in High-Fat Diet-Induced Obese Mice

**Figure 7:**
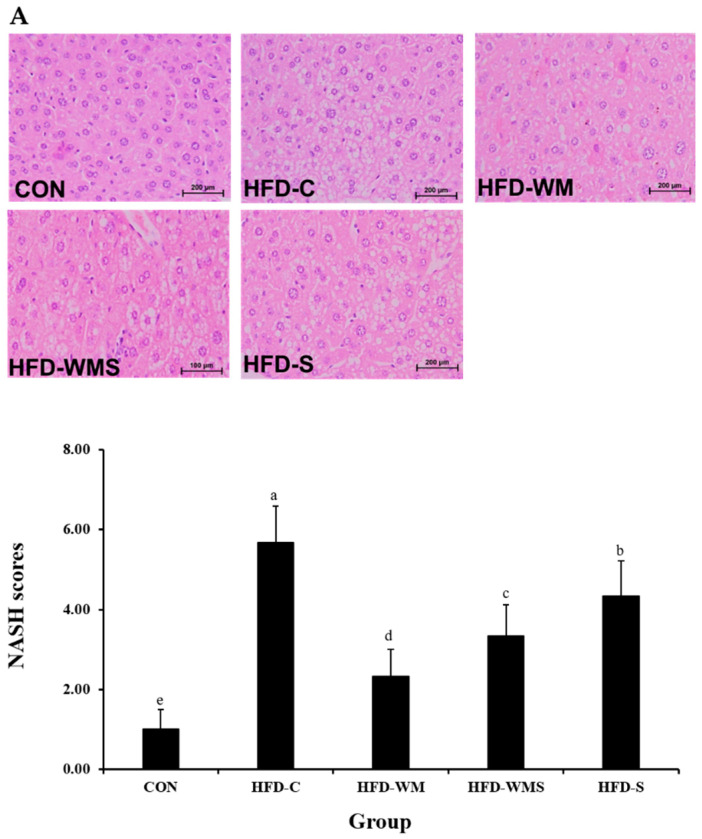

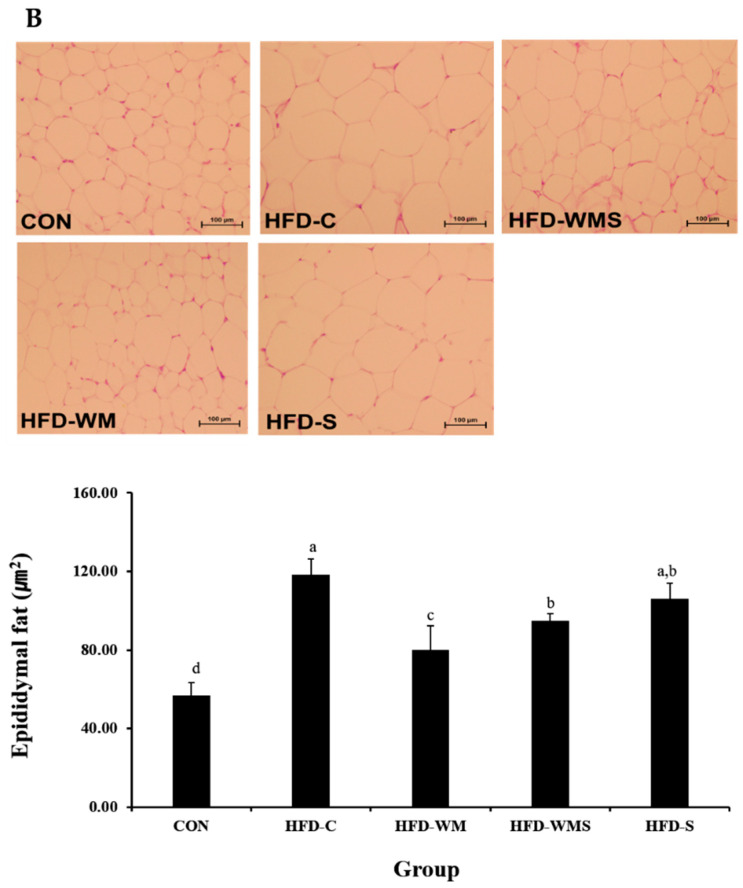
Effect of WM and WMS on histological (**A**) liver tissue and (**B**) epididymal adipocyte size in C57BL/6 mice obesity model induced by a high-fat diet. CON: Normal Diet + DW; HFD-C: High-Fat Diet (60%) + DW; HFD-WM: High-Fat Diet (60%) + WM 380 mg/kg; HFD-WMS: High-Fat Diet (60%) + WMS 380 mg/kg; HFD-S: High-Fat Diet (60%) + Salt. ^a–e^ Means with different superscripts are significantly different (*p* < 0.05) as analyzed using Duncan’s multiple range test.

Liver tissue evaluation through H&E staining and optical microscopy examination is presented in Figure 7A. In the CON group, assessment of steatosis grade, lobular inflammation, and hepatocellular ballooning revealed minimal fat accumulation, with no observable inflammation or ballooning. Hepatocytes maintained distinct round nuclei and relatively uniform spacing. HFD-C mice exhibited severe hepatic steatosis, inflammation, and ballooning, resulting in a 5.7-fold increase in NASH compared with the CON group. WM markedly improved these pathological features. The HFD-WM group showed 55.6% and 40.0% reductions in steatosis and lobular inflammation scores, respectively, with complete elimination of ballooning (100% reduction). NASH was reduced by 58.8% compared with HFD-C. The HFD-WMS group also demonstrated substantial improvements, with a 41.2% decrease in NASH. Epididymal adipocyte hypertrophy, which more than doubled in HFD-C mice, was reduced by WM 39.6% and WMS 22.8% (Figure 7B). These results indicate that WM effectively alleviates both hepatic injury and adipocyte enlargement induced by high-fat diet feeding.

## 4. Discussion

In this study, we investigated the anti-obesity effects and sodium-excreting action of immature watermelon salt, prepared by adding salt to an extract of immature watermelon, when orally administered to high-fat diet (HFD)-induced obese animal models. Obesity is a rapidly increasing chronic disease worldwide and is a major risk factor for cardiovascular disease, type 2 diabetes, and metabolic syndrome [40]. As such, there is growing interest in the development of safe and effective strategies for weight management [41]. Watermelon contains citrulline, which is known to have diuretic effects, and is rich in potassium, which facilitates sodium excretion [22]. Recent studies have focused on strategies to suppress obesity by preventing intracellular fat accumulation or stimulating the breakdown of stored fat [42]. L-citrulline, a non-essential amino acid, has drawn attention for its potential to enhance exercise performance and improve metabolism, thereby exerting anti-obesity effects [43]. The citrulline content of the immature watermelon extract (WM) and immature watermelon salt (WMS) used in this study was analyzed using HPLC, and the levels were found to be 7.7 mg/g and 3.1 mg/g, respectively. The HPLC–UV method used for citrulline quantification follows previously validated protocols demonstrating high linearity, repeatability, and recovery (RSD < 2%, recovery 95–103%) under identical chromatographic conditions [44,45]. Although full validation was not performed in our laboratory, our analytical parameters were consistent with these validated methods, supporting the accuracy of citrulline determination in WM and WMS. According to Rimando et al. [46], the citrulline content in watermelon rind with different flesh colors ranged from 0.8 to 1.50 mg/g, indicating that the immature watermelon used in this experiment contained a higher amount of citrulline. L-citrulline, abundantly present in watermelon rind, has been shown to increase mitochondrial fatty acid oxidation and up-regulate UCP1 expression in adipose tissue, thereby elevating energy expenditure [47]. Furthermore, watermelon rind extract may exert anti-inflammatory effects, improving adipose tissue homeostasis via increased adiponectin and reduced pro-inflammatory cytokines [48]. Indeed, recent studies in obese models have demonstrated that watermelon extract supplementation reduces inflammatory markers and improves lipid metabolism [49]. Although this study did not assess these mechanistic pathways directly, these observations provide a biological rationale for the observed reductions in fat mass and histological improvements in the liver. Future work should include gene- and protein-level assays to elucidate these mechanisms. Furthermore, Takeda et al. (2016) [50] reported in an animal study that citrulline supplementation improved mitochondrial function and promoted fatty acid oxidation. This suggests that citrulline may optimize cellular energy production processes, enhancing the use of fat as a primary energy source. In our experiment, the measurement of food intake in HFD-induced obese animals over a 6-week period showed that although the HFD-C and HFD-S groups did not exhibit significant weight reduction, the HFD-WM and HFD-WMS groups demonstrated a significant decrease in body weight. The slight reduction in feed intake observed in the WM and WMS groups, although not statistically significant per day, may cumulatively contribute to reduced caloric intake over the experimental period, thereby enhancing the anti-obesity effect. Also, the comparable efficacy between WM and WMS may result from matrix components (fibers, phenolics, minerals) influencing L-citrulline bioavailability and NO-related natriuresis. Overall, the main mechanistic focus was placed on the Na^+^/K^+^-ATPase-dependent regulation of fluid and energy homeostasis [51], whereas dietary salt and the citrulline contained in the immature watermelon rind extract were considered auxiliary modulators and markers that may act synergistically along this axis. In other words, the extract was not intended to isolate the effect of citrulline alone but rather to evaluate how a potassium- and citrulline-rich matrix could support Na^+^/K^+^-ATPase-mediated sodium handling, mitochondrial function, and energy expenditure, thereby exerting anti-obesity effects through an integrated and complementary mechanism. Future work will include an L-citrulline control and molecular assays. Upon necropsy, visible abdominal fat was observed in all groups except the control group (CON), with reduced fat accumulation observed in the HFD-WM and HFD-WMS groups compared to the HFD-C group. This finding is consistent with the study by Lee et al. [52], in which the administration of plant-based salt (Salicornia) significantly reduced body weight and weight gain in the HHS group compared to the HH control group. After six weeks of test substance administration and diet intervention, measurements of epididymal and retroperitoneal fat weights showed a reduction in retroperitoneal fat in the HFD-WM and HFD-WMS groups compared to the HFD-C group, and a significant decrease was observed relative to the CON group. Interestingly, several parameters (body weight, visceral fat mass, and NASH score) were slightly lower in the HFD-S group than in the HFD-C group, suggesting that high-salt supplementation alone may induce mild diuretic or osmotic effects [53]. However, WM and WMS supplementation produced more consistent and pronounced improvements than refined salt, indicating that rind-derived bioactive components, rather than sodium per se, contribute to the observed anti-obesity and hepatic benefits [54,55]. Although significant reductions in body and liver weights were observed, no OGTT or IPGTT was conducted. Consequently, this study cannot address whether WM or WMS improved glucose tolerance or insulin sensitivity. Future investigations should incorporate these metabolic tests to determine whether anti-obesity effects are accompanied by improved glucose regulation. Lipid metabolism is essential for energy storage and distribution, regulation of glucose metabolism, and maintenance of energy homeostasis [56]. Adipose tissue is heterogeneously distributed in multiple depots that differ in physiological function and metabolic risk. Visceral white adipose tissue (vWAT) is strongly linked to metabolic disease, whereas subcutaneous WAT (scWAT) is comparatively less harmful and may even exert protective effects [57,58]. Brown adipose tissue (BAT) dissipates energy via UCP1-mediated thermogenesis and is a recognized target for obesity intervention [59,60]. Notably, our study evaluated only liver and epididymal WAT (a rodent visceral depot) and did not examine scWAT or BAT. This limitation means that the metabolic impact of WM and WMS on other fat depots remains unknown. Future work should include assessments of scWAT, multiple visceral depots, and BAT to provide a more comprehensive understanding of the anti-obesity and metabolic effects of these extracts. Our findings align with evidence that habitual high sodium intake is associated with greater adiposity and central overweight/obesity in humans, and with emerging data linking high salt to visceral adipose dysfunction [55,61]. Hyperlipidemia, characterized by elevated levels of cholesterol, triglycerides, phospholipids, and free fatty acids in the blood, is a major cause of atherosclerosis, which underlies angina, stroke, aneurysms, and hypertension [62]. In this study, the levels of triglycerides and total cholesterol, key indicators of this condition, were lower in the HFD-WM and HFD-WMS groups compared to the HFD-C and HFD-S groups. Figueroa et al. (2019) [63] demonstrated that watermelon juice supplementation in postmenopausal women reduced arterial stiffness and improved vascular function. Improved vascular health enhances systemic circulation, promoting metabolic activity, and potentially supporting long-term weight management. In our results, serum sodium (Na^+^) levels were elevated by 43.2 ± 7.6% in the WM and WMS groups compared to the S group; however, the increase in the WMS group was significantly lower at 21.5 ± 6.6%. The diuretic and natriuretic potential of WM and WMS is supported by evidence that potassium and citrulline enhance renal sodium excretion through NO-mediated vasodilation, increased renal blood flow, and modulation of Na^+^/K^+^-ATPase-dependent tubular sodium transport. Although urinary Na^+^ and K^+^ excretion was not measured, the serum sodium profile observed in this study represents a complementary physiological indicator, as stabilization or reduction of serum sodium during sodium-containing extract supplementation suggests improved renal sodium handling and reduced extracellular fluid expansion. These findings are consistent with the known natriuretic mechanisms of potassium- and citrulline-rich foods. Also, their study showed that a low-sodium diet decreased serum triglyceride levels to 73.8 ± 12.5 mg/dL compared to 89.4 ± 27.3 mg/dL in the high-sodium group, which aligns with our findings regarding WMS administration. High sodium intake has been reported to increase plasma leptin levels and activate enzymes involved in lipogenesis, leading to adipocyte hypertrophy and visceral fat accumulation. These changes contribute to insulin resistance in adipose tissue, increasing the risk of type 2 diabetes and cardiovascular diseases [9,64,65]. Collins et al. (2017) [66] found that citrulline from watermelon extracts exhibited anti-inflammatory effects, contributing to improved metabolic function. Reducing inflammation is known to promote the secretion of beneficial adipokines such as adiponectin, thereby enhancing lipid metabolism. The present study examined a 6-week treatment period, which is relatively short for assessing long-term safety and efficacy. Thus, the durability of the anti-obesity effects of WM and WMS and their chronic safety profile remain unknown. Future research should include prolonged consumption trials, chronic toxicity assessments, and long-term follow-up to determine whether the benefits are sustained and safe. Recent systematic reviews of plant-derived anti-obesity agents underscore the necessity of such longer-term evaluations [67]. Currently, FDA-approved anti-obesity drugs include lipase inhibitors such as Orlistat and serotonin-norepinephrine reuptake inhibitors like Sibutramine. However, these drugs are associated with adverse effects, including steatorrhea, nausea, vomiting, headaches, constipation, insomnia, and gastrointestinal disturbances [68]. The results of this experiment are expected to reflect the citrulline and diuretic effects in WM and WMS, which contain acid salts. Although WM contained more than twice the citrulline content of WMS, their bioactivities were comparable. Although intestinal flora and metabolic signaling pathways were not directly assessed, the existing literature suggests mechanistic pathways consistent with our findings. Potassium-rich extracts may enhance renal Na^+^ excretion via Na^+^/K^+^-ATPase modulation, while citrulline stimulates NO-mediated mitochondrial fatty acid oxidation and increases ATP turnover. Additionally, plant-derived amino acids can influence gut microbial composition and reduce metabolic inflammation [69]. These mechanisms collectively provide theoretical support for the observed reductions in adiposity and lipid profiles. Future investigations will include an L-citrulline control group and examine gene/protein targets involved in citrulline pathways to better elucidate the mechanism. Despite the significant metabolic improvements observed in this study, several limitations should be acknowledged. First, the present findings are based on a high-fat diet-induced obesity model in mice, which, although widely used, does not fully recapitulate the complexity and heterogeneity of human obesity. Species-specific differences in metabolism, gut microbiota composition, sodium and potassium handling, and nitric oxide-related signaling pathways may influence the physiological responses to watermelon-derived extracts. Therefore, caution should be exercised when extrapolating these results directly to human physiology. Further studies, including well-designed clinical trials, are warranted to validate the translational relevance of the observed anti-obesity and metabolic effects in humans.

## 5. Conclusions

This study demonstrated that immature watermelon extract (WM) and watermelon salt (WMS), when administered to HFD-induced obese models, significantly reduced body weight, abdominal fat accumulation, retroperitoneal fat weight, and blood lipid levels, including triglycerides and total cholesterol. The increased sodium excretion observed in the WM and WMS groups, likely due to their citrulline and potassium content, may contribute to improved lipid metabolism. These results suggest that WM and WMS have potential as natural anti-obesity agents by promoting fat reduction and metabolic health.

## Figures and Tables

**Table 1:** Composition of experimental diets.

Component	Normal Diet		High-Fat Diet (60%)	
Product	(gm %)	(kcal %)	(gm %)	(kcal %)
Protein	19.2	20	26.2	20
Carbohydrate	67.3	70	26.3	20
Fat	4.3	10	34.9	60
Total		100		100
Ingredient	(gm)	(kcal)	(gm)	(kcal)
Casein, 80Mesh	200	800	200	800
L-Cystin	3	12	3	12
Corn Starch	315	1260	0	0
Maltodextrin 10	35	140	125	500
Sucrose	350	1400	68.8	275.2
Cellulose, BW200	50	0	50	0
Soybean Oil	25	225	25	225
Lard	20	180	245	2205
Mineral Mix S10026	10	0	10	0
DiCalcium Phosphate	13	0	13	0
Calcium Carbonate	5.5	0	5.5	0
Potassium Citrate, 1 H20	16.5	0	16.5	0
Vitamin Mix V10001	10	40	10	40
Choline Bitartrate	2	0	2	0
FD&C Yellow Dye#5	0.05	0	0	0
FD&C Blue Dye#1	0	0	0.05	0
Total	1055.05	4057	773.85	4057

**Table 2:** Experimental design for a six-week animal study on the anti-obesity effects of WMS.

Group		Material	Dose	*n*
1	CON	Normal Diet + DW	-	5
2	HFD-C	High-Fat Diet + DW	-	5
3	HFD-WM	High-Fat Diet + WM	380 mg/kg/day	5
4	HFD-WMS	High-Fat Diet + WMS	380 mg/kg/day	5
5	HFD-S	High-Fat Diet + Salt	380 mg/kg/day	5

**Table 3:** Anti-obesity effect of WM and WMS on body weight gain and feed efficiency ratio of mice fed high-fat-supplemented diets for 6 weeks.

Measurements	CON	HFD-C	HFD-WM	HFD-WMS	HFD-S
Initial body weight (g)	20.06	19.28	19.00	18.82	18.88
Final body weight (g)	30.58	43.22	33.78	35.88	39.72
Feed intakes (g/day)	28.38	24.44	22.96	23.24	24.26
Water intakes (mL/day)	32.30	23.24	22.18	30.49	23.49
Weight gain (g/6 week)	10.52	23.94	14.96	16.88	20.84
FER	0.01	0.02	0.02	0.02	0.02

CON: Normal Diet + DW; HFD-C: High-Fat Diet (60%) + DW; HFD-WM: High-Fat Diet (60%) + WM 380 mg/kg; HFD-WMS: High-Fat Diet (60%) + WMS 380 mg/kg; HFD-S: High-Fat Diet (60%) + Salt.

**Table 4:** Effect of serum electronic ion level on WM and WMS.

Measurement		Group			
		DW	S	WM	WMS
Serum sodium (mmol/L)	0 min	85.0 ± 5.2	95.0 ± 10.8	82.7 ± 21.4	95.7 ± 14.7
	60 min	101 ± 2.6	131.0 ± 2.0	99.0 ± 10.1	111.7 ± 8.7

## Data Availability

The original contributions presented in this study are included in the article. Further inquiries can be directed to the corresponding author.

## References

[1] Mokdad A.H., Bowman B.A., Ford E.S., Vinicor F., Marks J.S., Koplan J.P. (2001). The continuing epidemics of obesity and diabetes in the United States. JAMA.

[2] World Health Organization The Statistics of Obesity. https://www.who.int/health-topics/obesity#tab=tab_1.

[3] Cardel M.I., Atkinson M.A., Taveras E.M., Holm J.C., Kelly A.S. (2020). Obesity treatment among adolescents: A review of current evidence and future directions. JAMA Pediatr..

[4] Bray G.A. (2004). Medical consequences of obesity. J. Clin. Endocrinol. Metab..

[5] Sharma A.M. (2002). Adipose tissue: A mediator of cardiovascular risk. Int. J. Obes..

[6] Björntorp P. (1988). The associations between obesity, adipose tissue distribution and disease. Acta Med. Scand. Suppl..

[7] Na S.Y., Myung S.J. (2012). Obesity and colorectal cancer. Korean J. Gastroenterol..

[8] Kyung M.G., Lim J.Y., Lee K.S., Jung S.W., Choe K.B., Yang C.K., Kim Y.R. (2014). Effects of short-term supplementation of erythritol-salt on urinary electrolyte excretion in rats. J. Nutr. Health.

[9] Fonseca-Alaniz M.H., Brito L.C., Borges-Silva C.N., Takada J., Andreotti S., Lima F.B. (2007). High dietary sodium intake increases white adipose tissue mass and plasma leptin in rats. Obesity.

[10] Park J.S., Akbar H., Yim J.-E. (2024). Correlation between sodium intake and obesity with related factors among Koreans: A cross-sectional study on dietary intake and eating habits. J. Nutr. Health.

[11] Ministry for Health, Welfare and Family Affairs, Korea Centers for Disease Control and Prevention (2012). National Health Nutrition Examination Survey Report.

[12] Choi J., Moon H.K. (2010). Nutrients and dish intake by fasting blood glucose level. Korean J. Nutr..

[13] Yon M., Lee Y., Kim D., Lee J., Koh E., Nam E., Shin H., Kang B.W., Kim J.W., Heo S. (2011). Major sources of sodium intake of the Korean population at prepared dish level: Based on the KNHANES 2008 & 2009. Korean J. Community Nutr..

[14] Kang J.G., Park C.Y. (2012). Anti-obesity drugs: A review about their effects and safety. Diabetes Metab. J..

[15] Kim K.S., Park S.W. (2012). Drug therapy for obesity. Korean J. Obes..

[16] Wang Y.M., van Eys J. (1981). Nutritional significance of fructose and sugar alcohols. Annu. Rev. Nutr..

[17] Hong S.P., Lim J.Y., Jeong E.J., Shin D.H. (2008). Physicochemical properties of watermelon according to cultivars. Korean J. Food Preserv..

[18] Kim K.S., Lee H.J., Kim S.M. (1999). Volatile flavor components in watermelon and oriental melon. Korean J. Food Sci. Technol..

[19] Hwang Y., Lee K.K., Jung G.T., Ko B.R., Choi D.C., Choi J.S., Eun J.B. (2004). Manufacturing of watermelon beverage added with natural color extracts. Korean J. Food Sci. Technol..

[20] Hwang Y., Lee K.K., Jung G.T., Ko B.R., Choi D.C., Choi Y.G., Eun J.B. (2004). Manufacturing of wine with watermelon. Korean J. Food Sci. Technol..

[21] Suh J.Y., Kang H.A., Chang K.S. (2001). Concentration of watermelon juice by reverse osmosis. Food Eng. Prog..

[22] Sohn J.Y., Ban S.C., Shin J.S., Hong S.H. (1996). Distribution of free sugars in the various portions of watermelon (*Citrullus vuigaris* L.) and muskmelon (*Cucumis meio* var. reticulatus Naud.). Appl. Biol. Chem..

[23] Kikuchi T., Ikedaya A., Toda A., Ikushima K., Yamakawa T., Okada R., Yamada T., Tanaka R. (2015). Pyrazole alkaloids from watermelon (*Citrullus lanatus*) seeds. Phytochem. Lett..

[24] Madhavi P., Rao M., Vakati K., Rahman H., Eswaraiah M.C. (2012). Evaluation of anti-inflammatory activity of *Citrullus lanatus* Seed Oil by In-vivo and In-vitro Models. Int. J. Pharm. Sci. Rev. Res..

[25] Siddig I.A., Loiy E.A.H., Hasnah M.S., Sakina M.A.Y., Waleed S.K., Syam M., Manal M.E.T., Syahida A., Cheah S.C., Putri N. (2011). Anti-inflammatory activities of cucurbitacin E isolated from *Citrullus lanatus* var. *citroides*: Role of reactive nitrogen species and cyclooxygenase enzyme inhibition. Fitoterapia.

[26] Kang H.M., Park S.Y., Kim J.E., Lee K.W., Hwang D.Y., Choi Y.W. (2024). *Citrullus mucosospermus* Extract Reduces Weight Gain in High-Fat Diet-Induced Obese C57BL/6N Mice. Nutrients.

[27] Daughtry J., Rasmussen C., Rosas M., Zhang L., Lu S., Hooshmand S., Liu C., Kern M., Hong M.Y. (2023). Blenderized watermelon consumption decreases body mass index, body mass index percentile, body fat and HbA1c in children with overweight or obesity. Pediatr. Obes..

[28] Baião D.D.S., Da Silva D.V.T., Paschoalin V.M.F. (2025). Watermelon Nutritional Composition with a Focus on L-Citrulline and Its Cardioprotective Health Effects—A Narrative Review. Nutrients.

[29] Volino-Souza M., Foureaux G., dos Santos A.G., Rocha K.S., Cella P.S., da Mota M.M.P., da Costa D.P.B., Lins E.S.D.S., de Carvalho T.F., da Silva J.J.S. (2022). Current Evidence of Watermelon (*Citrullus lanatus*) Ingestion on Vascular Health: A Food Science and Technology Perspective. Nutrients.

[30] Arulselvan P., Senthilkumar G.P., Kaur J., Sreepriya M., Thangam E.B., Somasundaram S. (2012). Dietary administration of scallion extract effectively inhibits colorectal tumor growth: Cellular and molecular mechanisms in mice. PLoS ONE.

[31] Benkeblia N., Shiomi N. (2006). Fructooligosaccharides of edible alliums: Occurrence, chemistry and health benefits. Curr. Nutr. Food Sci..

[32] He F.J., MacGregor G.A. (2010). Reducing population salt intake worldwide: From evidence to implementation. Prog. Cardiovasc. Dis..

[33] Bahri S., Curis E., El Wafi F., Cerutti C., Crenn P., Chaumeil J.-C., Cynober L., Béziel K. (2008). Mechanisms and kinetics of citrulline uptake in a model of human intestinal epithelial cells. Clin. Nutr..

[34] Moinard C., Nicolis I., Neveux N., Darquy S., Benazeth S., Cynober L. (2008). Dose-ranging effects of citrulline administration on plasma amino acids and hormonal patterns in healthy subjects: The Citrudose pharmacokinetic study. Br. J. Nutr..

[35] Joffin N., Jaubert A.-M., Bamba J., Barouki R., Noirez P., Forest C. (2015). Acute induction of uncoupling protein 1 by citrulline in cultured explants of white adipose tissue from lean and high-fat-diet-fed rats. Adipocyte.

[36] Joffin N., Jaubert A.-M., Durant S., Bastin J., De Bandt J.-P., Cynober L., Moinard C., Coumoul X., Forest C., Noirez P. (2015). Citrulline reduces glyceroneogenesis and induces fatty acid release in visceral adipose tissue from overweight rats. Mol. Nutr. Food Res..

[37] He F.J., MacGregor G.A. (2009). A comprehensive review on salt and health and current experience of worldwide salt reduction programmes. J. Hum. Hypertens..

[38] Nair A.B., Jacob S. (2016). A simple practice guide for dose conversion between animals and human. J. Basic Clin. Pharm..

[39] Neuenschwander B., Capkun-Niggli G., Branson M., Spiegelhalter D.J. (2010). Summarizing historical information on controls in clinical trials. Clin. Trials.

[40] Afshin A., Forouzanfar M.H., Reitsma M.B., Sur P., Estep K., Lee A., Marczak L., Mokdad A.H., Moradi-Lakeh M., Naghavi M. (2017). Health effects of overweight and obesity in 195 countries over 25 years. N. Engl. J. Med..

[41] Wadden T.A., Butryn M.L., Hong P.S., Tsai A.G. (2014). Behavioral treatment of obesity in patients encountered in primary care settings: A systematic review. JAMA.

[42] Cha S.Y., Jang J.Y., Lee Y.H., Lee G.Y., Lee H.J., Hwang K.T., Kim Y.J., Jun Y.J., Lee J.M. (2010). Lipolytic effect of methanol extracts from *Luffa cylindrica* in mature 3T3-L1 adipocytes. J. Korean Soc. Food Sci. Nutr..

[43] Curis E., Nicolis I., Moinard C., Osowska S., Zerrouk N., Bénazeth S., Cynober L. (2005). Almost all about citrulline in mammals. Amino Acids.

[44] Wu G., Meininger C.J. (2002). Regulation of nitric oxide synthesis by dietary factors. Annu. Rev. Nutr..

[45] Causon R.C., Carruthers M.E. (1982). Measurement of catecholamines in biological fluids by high-performance liquid chromatography: A comparison of fluorimetric with electrochemical detection. J. Chromatogr..

[46] Rimando A.M., Perkins-Veazie P.M. (2005). Determination of citrulline of watermelon rind. J. Chromatogr. A.

[47] Allerton T.D., Proctor D.N., Stephens J.M. (2018). _L_-Citrulline Supplementation: Impact on Cardiometabolic Health. Nutrients.

[48] Guo L., Park S.Y., Kang H.M., Kang N.J., Hwang D.Y., Choi Y.-W. (2022). Edible Vitalmelon Fruit Extract Inhibits Adipogenesis and Ameliorates High-Fat Diet-Induced Obesity. Biomed Res. Int..

[49] Miyai S., Hashizume T., Okazaki T. (2018). Effects of a Watermelon Extract Beverage on Canine Lipid Metabolism and Urine Crystals. Anim. Vet. Sci..

[50] Takeda K., Machida M., Kohara A., Omi N., Takemasa T. (2016). Effects of citrulline supplementation on fatigue and exercise performance in mice. J. Nutr. Biochem..

[51] Blanco G., Mercer R.W. (1998). Isozymes of the Na-K-ATPase: Heterogeneity in structure, diversity in function. Am. J. Physiol.-Cell Physiol..

[52] Lee H.S., Choi J.H., Kim Y.E., Lee C.H. (2012). Effect of dietary intake of *Salicornia herbacea* L. hot water extract on anti-obesity in diet-induced obese rats. J. Korean Soc. Food Sci. Nutr..

[53] Drenjančević-Perić I., Jelaković B., Lombard J.H., Kunert M.P., Kibel A., Gros M. (2010). High-salt diet and hypertension: Focus on the renin–angiotensin system. Kidney Blood Press. Res..

[54] Becraft A.R., Sturm M.L., Mendez R.L., Park S.H., Lee S.I., Shay N.F. (2020). Intake of watermelon or its byproducts alters glucose metabolism, the microbiome, and hepatic proinflammatory metabolites in high-fatfed male C57BL/6 J mice. J. Nutr..

[55] Wu Q., Burley G., Li L.C., Lin S., Shi Y.C. (2023). The role of dietary salt in metabolism and energy balance: Insights beyond cardiovascular disease. Diabetes Obes. Metab..

[56] Jeong H.S., Jeong J.C. (2010). Anti-adipogencic effect of Piper nigrum Linne. Korean J. Orient. Physiol. Pathol..

[57] An S.M., Kim J.-H. (2023). Adipose tissue and metabolic health. Diabetes Metab. J..

[58] Zotti T., Giacco A., Cuomo A., Cerulo L., Petito G., Iervolino S., Roperto A.P., Gnoni A., Gnoni G.V., Cioffi F. (2023). Exercise equals the mobilization of visceral versus subcutaneous adipose fatty acid molecules in fasted rats associated with modulation of the AMPK/ATGL/HSL axis. Nutrients.

[59] Shinde A.B., Song A., Wang Q.A. (2021). Brown adipose tissue heterogeneity, energy metabolism, and beyond. Front. Endocrinol..

[60] Carpentier A.C., Blondin D.P., Nedergaard J. (2023). Brown adipose tissue—A translational perspective. Endocr. Rev..

[61] Zhou X., Chen Z., Yun X., Chen S., Jiang H., Chen W., Lin L. (2014). High-salt intake induced visceral adipose tissue hypoxia and its association with circulating monocyte subsets in humans. Am. J. Clin. Nutr..

[62] Shin H.S., Kim G.Y., Seo I.B., Kim H.H. (2003). Preventive effects of Typhae pollen on the diet-induced hyperlipidemia in rats. J. Korean Orient. Med..

[63] Figueroa A., Wong A., Kalfon R., Hooshmand S., Sanchez-Gonzalez M.A. (2019). Effects of watermelon supplementation on arterial stiffness and wave reflection amplitude in postmenopausal women. Menopause.

[64] Fonseca-Alaniz M.H., Takada J., Andreotti S., de Campos T.B., Campaña A.B., Borges-Silva C.N., Lima F.B. (2008). High sodium intake enhances insulin-stimulated glucose uptake in rat epididymal adipose tissue. Obesity.

[65] Neeland I.J., Turer A.T., Ayers C.R., Powell-Wiley T.M., Vega G.L., Farzaneh-Far R., Grundy S.M., Khera A., McGuire D.K., de Lemos J.A. (2012). Dysfunctional adiposity and the risk of prediabetes and type 2 diabetes in obese adults. JAMA.

[66] Collins J.K., Wu G., Perkins-Veazie P., Spears K., Claypool P.L., Baker R.A., Clevidence B.A. (2017). Watermelon consumption increases plasma arginine concentrations in adults. Food Funct..

[67] Shaik Mohamed Sayed U.F., Moshawih S., Goh H.P., Kifli N., Gupta G., Singh S.K., Chellappan D.K., Dua K., Hermansyah A., Ser H.L. (2023). Natural products as novel anti-obesity agents: Insights into mechanisms of action and potential for therapeutic management. Front. Pharmacol..

[68] Min O.J., Sharma B.R., Park C.M., Rhyu D.Y. (2011). Effect of Myadis stigma water extract on adipogenesis and blood glucose in 3T3-L1 adipocytes and db/db mice. Korean J. Pharmacogn..

[69] Beaumont M., Portune K.J., Steuer N., Lan A., Cerrudo V., Audebert M., Dumont F., Mancano G., Khodorova N., Andriamihaja M. (2022). Selective nourishing of gut microbiota with amino acids: A novel prebiotic approach?. Front. Nutr..

